# Use of the Wasserman equation in optimization of the duration of the power ramp in a cardiopulmonary exercise test: a study of Brazilian men

**DOI:** 10.1590/1414-431X20154692

**Published:** 2015-09-18

**Authors:** D. C. Costa, G. L. de Santi, J. C. Crescêncio, L. P. Seabra, E. E. V. Carvalho, V. Papa, F. Marques, L. Gallo, A. Schmidt

**Affiliations:** Laboratório de Fisiologia do Exercício, Divisão de Cardiologia, Departamento de Clínica Médica, Hospital das Clínicas, Faculdade de Medicina de Ribeirão Preto, Universidade de São Paulo, Ribeirão Preto, SP, Brasil

**Keywords:** Wasserman equation, Cardiopulmonary exercise test, Coronary artery disease, Cardiovascular diagnostic techniques

## Abstract

This study aimed to analyze the agreement between measurements of unloaded oxygen uptake and peak oxygen uptake based on equations proposed by Wasserman and on real measurements directly obtained with the ergospirometry system. We performed an incremental cardiopulmonary exercise test (CPET), which was applied to two groups of sedentary male subjects: one apparently healthy group (HG, n=12) and the other had stable coronary artery disease (n=16). The mean age in the HG was 47±4 years and that in the coronary artery disease group (CG) was 57±8 years. Both groups performed CPET on a cycle ergometer with a ramp-type protocol at an intensity that was calculated according to the Wasserman equation. In the HG, there was no significant difference between measurements predicted by the formula and real measurements obtained in CPET in the unloaded condition. However, at peak effort, a significant difference was observed between oxygen uptake ($\dot{\text{V}}$O_2_)_peak(predicted)_and $\dot{\text{V}}$O_2peak(real)_(nonparametric Wilcoxon test). In the CG, there was a significant difference of 116.26 mL/min between the predicted values by the formula and the real values obtained in the unloaded condition. A significant difference in peak effort was found, where $\dot{\text{V}}$O_2peak(real)_was 40% lower than $\dot{\text{V}}$O_2peak(predicted)_(nonparametric Wilcoxon test). There was no agreement between the real and predicted measurements as analyzed by Lin’s coefficient or the Bland and Altman model. The Wasserman formula does not appear to be appropriate for prediction of functional capacity of volunteers. Therefore, this formula cannot precisely predict the increase in power in incremental CPET on a cycle ergometer.

## Introduction

The cardiopulmonary exercise test (CPET) has greatly changed the approach to functional evaluation by relating physical fitness and physiological parameters to the underlying metabolic substrate and by providing highly reproducible descriptors of effort capacity. The CPET provides an accurate and objective measurement of functional capacity and of the integrity of the cardiovascular and respiratory systems. Therefore, the CPET has been indicated for assessment of functional capacity in high-performance athletes, for diagnostic purposes, and for evaluation of pharmacological or non-pharmacological therapies, preoperative risk, and for risk stratification. Variables, such as oxygen uptake ($\dot{\text{V}}$O_2_) represent an objective measure of functional capacity and reflect the severity of diseases, such as myocardial ischemia, heart failure, hypertrophic cardiomyopathy, and pulmonary artery hypertension secondary to interstitial or chronic obstructive pulmonary disease (1,2).

The CPET is considered to be the most accurate method for assessment of aerobic power. However, different values may be obtained for the same individual when different modalities of the test are used (3,4). From a methodological point of view, two aspects crucially interfere with the quality of the test and the reproducibility of the response of the variables: the type of protocol and the duration of the effort tests.

Ramp protocols have become popular because they permit individualized tests. This is possible because load increment occurs constantly and continuously at a rate that can be individualized according to the capacity of the individual (5-8).

The Wasserman equation (9) has been routinely used for many years in the Laboratório de Fisiologia do Exercício, Faculdade de Medicina de Ribeirão Preto. This equation is used for the choice of progressive load increment on a cycle ergometer during the CPET, and is applied to healthy individuals and to those with ischemic myocardial disease. This practice is based on the search for a less empirical and subjective manner of choosing the intensity of the power ramp to be applied to incremental effort tests on a cycle ergometer.

Elaboration of a formula for the calculation of load increment in a ramp-type incremental CPET by Wasserman et al. (9) proved to be helpful for choosing the most appropriate intensity for optimization of the quality of the CPET. However, in many cases, a simple estimate of the power increment according to this formula can underestimate or overestimate the real functional capacity of a healthy individual or a patient. Marked characteristics of health status, as well as disease status, directly interfere with the performance and homeostasis of the cardiopulmonary and musculoskeletal systems during exercise.

The present study aimed to analyze the agreement between measurements of unloaded oxygen uptake ($\dot{\text{V}}$O_2unloaded(predicted)_) and peak oxygen uptake ($\dot{\text{V}}$O_2peak(predicted)_) predicted from the equations proposed by Wasserman and real measurements ($\dot{\text{V}}$O_2unloaded(real)_,$\dot{\text{V}}$O_2peak(real)_), which were directly obtained in an incremental ramp-type CPET. We studied a sample of apparently healthy sedentary males and a sample of sedentary males with coronary artery disease (CAD).

## Material and Methods

Two groups of sedentary males were selected. The healthy group (HG) consisted of 12 apparently healthy sedentary volunteers, aged 47±4 years, who were recruited through the media. For inclusion in the study, all of the subjects were submitted to clinical evaluation and to a resting electrocardiogram to exclude asymptomatic heart disease or a history of cardiac or pulmonary disease or any orthopedic limitation. Current smokers and/or alcoholics, and subjects taking antihypertensive drugs or beta-blockers, or any other medication that would alter the response of the subject to physical effort were excluded.

The other group was the coronary artery disease group (CG). This group consisted of 16 sedentary individuals, aged 57±8 years, who were recruited at the outpatient clinic of the institution. The subjects met at least one of the following inclusion criteria: a history of acute myocardial infarction that was clinically confirmed and/or by complementary exams, previous coronary angiography with documentation of coronary injury with more than 70% obstruction of the luminal diameter, and a previous noninvasive exam (stress echocardiogram or myocardial perfusion scintigraphy) with documentation of effort ischemia. All of the patients had been clinically stable for at least the last 3 months, with optimized pharmacological treatment and no indication of new revascularization procedures.

The study was approved by the Research Ethics Committee of the Hospital das Clínicas, Faculdade de Medicina de Ribeirão Preto, Universidade de São Paulo, (protocol #1593/2009). All of the subjects gave written informed consent to participate.

### CPET

The formula proposed by Wasserman (9) was calculated for each individual for performance of the CPET as follows:

$\dot{\text{V}}$O_2unloaded_(mL/min) = 150 + (6× weight, kg)

$\dot{\text{V}}$O_2peak_(mL/min) = (height: cm, age: years)×20

Ramp (w/min) = $\dot{\text{V}}$O_2peak_− $\dot{\text{V}}$O_2unloaded_/100

For subjects in the HG, only a decimal approximation of the final result of the formula, in watts/minute, was added by rounding the value to the nearest decimal point. For subjects in the CG, this correction was made based on clinical evaluation and disease conditions, with an average 4.84 watts reduction of the final result. These corrections needed to be applied so that each individual achieved maximum effort, satisfying one or more of the criteria that define $\dot{\text{V}}$O_2max_, such as a respiratory exchange ratio (RER) >1.15, heart rate at peak effort above 85% of maximum, and a plateau of oxygen uptake, which was defined as a variation in increase between 1.0 and 2.0 mL/kg/min within 8 to 12 min (3,7,10).

All of the subjects were submitted to a maximal CPET using a ramp-type incremental protocol. The individuals performed dynamic physical effort in the sitting position on an electromagnetically braked cycle ergometer (Corival 400, Lode BV, The Netherlands). The subjects were encouraged to perform applied effort up to the power at which they reached cardiorespiratory exhaustion and/or the presence of classical interruption criteria as follows: elevation of diastolic blood pressure to 120 mmHg in normotensive subjects and up to 140 mmHg in hypertensive subjects; marked systolic blood pressure elevation up to 260 mmHg; and exacerbated chest discomfort with increased load or associated with electrocardiographic changes, such as ischemia, ataxia, dizziness, pallor, cyanosis and presyncope, disproportional dyspnea, complex ventricular arrhythmia, and others (1,8). For all of the subjects, the beginning of the ramp exercise was preceded by 4 min of unloaded effort (3-4 watts) at a constant speed of 60 rotations per minute (rpm). In this protocol, the ventilatory variables were determined using the CPX/D metabolic analysis system (Medical Graphics, USA), which permits the acquisition, processing, and storage of breath-to-breath data. Laboratory conditions were set at 45% to 60% air relative humidity and an ambient temperature of approximately 22°C.

### Calculation of variables obtained with CPET

After stabilization of pedaling velocity at approximately 60 rpm, a segment of approximately 2 min was selected for calculation of $\dot{\text{V}}$O_2unloaded(real)_during the 4 min of the unloaded period. The last 10 values plotted by the metabolic system were selected for peak effort and the mean $\dot{\text{V}}$O_2peak(real)_was calculated.

### Statistical analysis

Data are reported as mean±SD and are presented graphically in box plots. The nonparametric Wilcoxon test was used to compare intragroup means of $\dot{\text{V}}$O_2unloaded(predicted)_, $\dot{\text{V}}$O_2unloaded(real)_, $\dot{\text{V}}$O_2peak(predicted)_, and $\dot{\text{V}}$O_2peak(real)_. The nonparametric Mann-Whitney test was used to compare intergroup means for duration of effort, $\dot{\text{V}}$O_2unloaded(real)_, $\dot{\text{V}}$O_2peak(real)_, RER unloaded, RER peak, power (peak), and ventilation ($\dot{\text{V}}$) peak. The agreement between means was determined using the Lin and Bland and Altman models, with calculation of the mean difference between methods (bias) and the 95% confidence intervals (CIs) for the limits of agreement.

Lin’s concordance coefficient was used to determine whether the measurements that were predicted by the formula and the real measurements that were obtained in the CPET significantly deviated from the perfect concordance line (a 45° line with origin at 0 of the x and y axes). Excellent concordance was defined as Lin >0.90, satisfactory concordance as Lin 0.6-0.9, and unsatisfactory concordance as Lin <0.6. The level of significance was set at 5%.

## Results

The anthropometric characteristics and risks factors of both groups are shown in Table 1. Previous events and interventions, distribution of the left ventricular ejection fraction, and medications used are shown in Table 2.

**Table:**
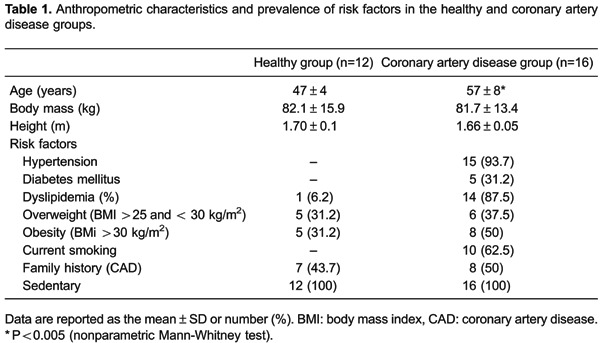

**Table:**
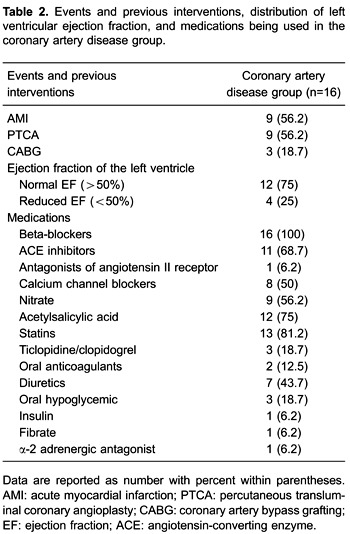

The duration of effort was 620±140 s in the CG, corresponding to 10±2 min, and 552±75 s in the HG, corresponding to 9±1 min, with no significant difference between the groups (P=0.09).

**Figure 1:**
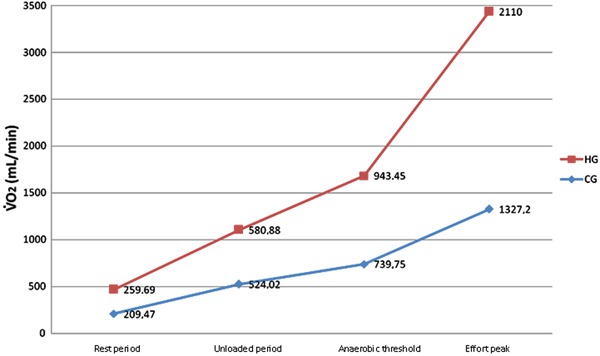
$\dot{\text{V}}$O_2_under conditions of rest and effort. $\dot{\text{V}}$O_2_was directly obtained by a metabolic analysis system during the CPET in the healthy group (HG) and coronary artery disease group (CG). $\dot{\text{V}}$O_2_: oxygen uptake.

With regard to the functional capacity of the two groups, during the unloaded period, $\dot{\text{V}}$O_2unloaded(real)_was significantly lower in the CG compared with the HG (524±67 *vs* 581±68 mL/min; P=0.02). At peak effort, $\dot{\text{V}}$O_2peak(real)_was significantly lower in the CG compared with the HG (1327±287 *vs* 2110±336 mL/min; P<0.0001). A similar finding was observed when $\dot{\text{V}}$O_2peak(real)_was corrected for weight (mL·kg^−1^·min^−1^), with a lower value in the CG compared with the HG (16±3 *vs* 26±6; P<0.0001; Figure 1 and Table 3).

**Table:**
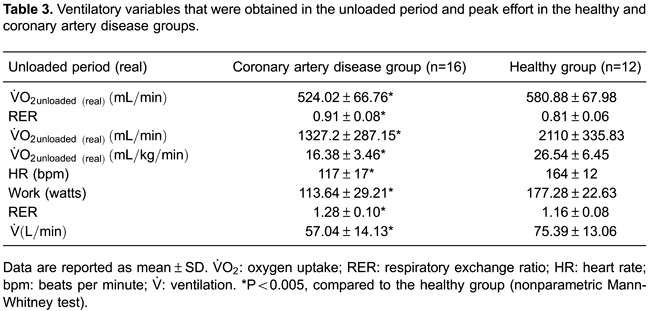

**Figure 2:**
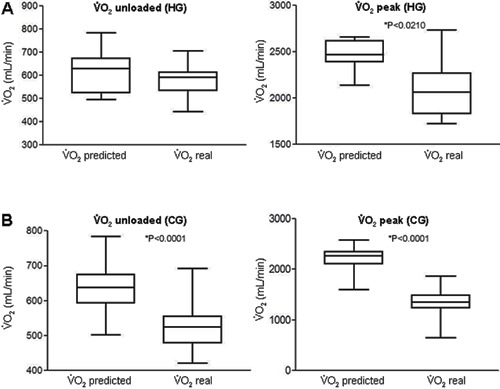
Box plots of $\dot{\text{V}}$O_2unloaded (predicted)_, $\dot{\text{V}}$O_2unloaded(real)_, $\dot{\text{V}}$O_2peak(predicted)_and $\dot{\text{V}}$O_2peak (real)_ in the healthy group (HG) (*A*) and the coronary artery disease group (CG) (*B*). The nonparametric Wilcoxon test was used for statistical analysis. $\dot{\text{V}}$O_2_: oxygen uptake.

When the measurements that were predicted by the formula and the real measurements that were obtained in the CPET were compared in the HG, the difference of 39 mL/min detected in the unloaded condition between $\dot{\text{V}}$O_2unloaded(predicted)_and $\dot{\text{V}}$O_2unloaded(real)_(620±91 *vs* 581±68 mL/min [6% difference]) was not significant (P=0.10). However, at peak effort, a significant difference was observed between $\dot{\text{V}}$O_2peak_(predicted) and $\dot{\text{V}}$O_2peak(real)_values (2467±174 *vs* 2110±336 mL/min [14% difference]; P=0.02; Figure 2A).

In the CG, there was a significant difference between the values that were predicted by the Wasserman (9) formula and the real values that were obtained in the unloaded condition in the CPET (116 mL/min, P<0.0001), where $\dot{\text{V}}$O_2unloaded(predicted)_was 640±80 mL/min and $\dot{\text{V}}$O_2unloaded(real)_was 524±67 mL/min (18% difference). A significant difference was also detected at peak effort, where $\dot{\text{V}}$O_2peak(predicted)_was 2217±241 and $\dot{\text{V}}$O_2peak(real)_was 1327±287 mL/min (P<0.0001; 40% difference; Figure 2B).

**Figure 3:**
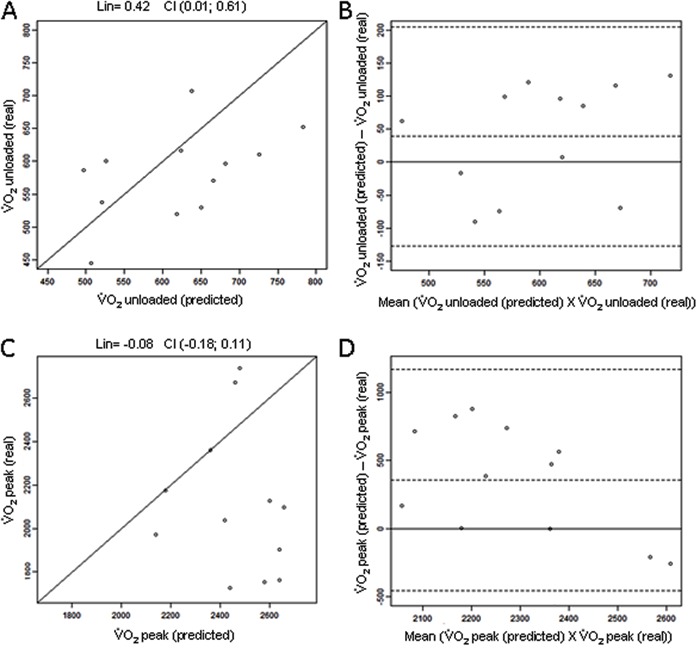
Lin’s concordance coefficients and Bland and Altman analysis in the healthy group. Lin’s concordance coefficient for $\dot{\text{V}}$O_2unloaded (predicted)_vs<$><$>$\dot{\text{V}}$O_2unloaded(real)_(A) and Bland and Altman analysis of $\dot{\text{V}}$O_2unloaded(predicted)_vs$\dot{\text{V}}$O_2unloaded(real)_(B) in the unloaded condition. Lin’s concordance coefficient for ($\dot{\text{V}}$O_2peak(predicted)_vs$\dot{\text{V}}$O_2unloaded (real)_)(C) and Bland and Altman analysis of $\dot{\text{V}}$O_2peak(predicted)_and<$><$>$\dot{\text{V}}$O_2unloaded (real)_(D) at peak effort. $\dot{\text{V}}$O_2_: oxygen uptake. Dotted lines: mean±2SD.

Analysis of agreement between the methods during the unloaded period in the HG showed a Lin’s concordance coefficient of 0.42 (95% CI=0.01-0.61) between the values that were predicted by the formula and the real values that were obtained in the CPET (Figure 3A). According to the Bland and Altman model, the mean difference ($\dot{\text{V}}$O_2unloaded(predicted)_−$\dot{\text{V}}$O_2unloaded(real)_) was 39 mL/min (95% CI=-127.08-204.92; Figure 3B). At peak effort, Lin’s concordance coefficient was 0.08 (95% CI=-0.18-0.11; Figure 3C). According to the Bland and Altman model, the mean difference ($\dot{\text{V}}$O_2peak(predicted)_−$\dot{\text{V}}$O_2unloaded(real)_) was 357 mL/min (95% CI=-454.82-1168.07; Figure 3D).

**Figure 4:**
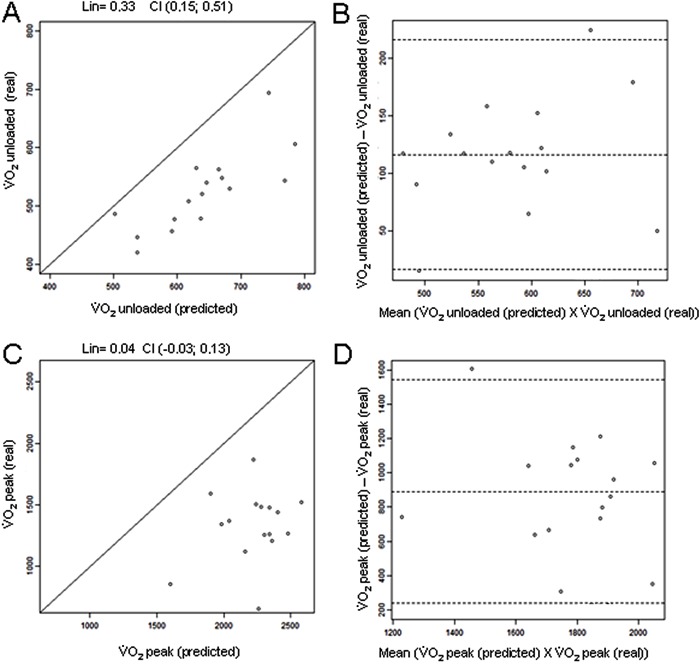
Lin’s concordance coefficients and Bland and Altman analysis in the coronary artery disease group. Lin’s concordance coefficient for $\dot{\text{V}}$O_2unloaded (predicted)_vs$\dot{\text{V}}$O_2unloaded (real)_(A) and Bland and Altman analysis of $\dot{\text{V}}$O_2unloaded (predicted)_vs<$><$>$\dot{\text{V}}$O_2unloaded (real)_(B) in the unloaded condition. Lin’s concordance coefficient for ($\dot{\text{V}}$O_2peak (predicted)_vs<$><$>$\dot{\text{V}}$O_2unloaded(real)_)(C) and Bland and Altman analysis of $\dot{\text{V}}$O_2peak (predicted)_and$\dot{\text{V}}$O_2unloaded(real)_(D) at peak effort. $\dot{\text{V}}$O_2_: oxygen uptake. Dotted lines: mean±2SD.

In contrast, in the CG, during the unloaded condition, Lin’s concordance coefficient between the values that were predicted by the formula and the real values that were obtained in the CPET was 0.33 (95% CI=0.15-0.51; Figure 4A). According to the Bland and Altman model, the mean difference ($\dot{\text{V}}$O_2unloaded(predicted)_−$\dot{\text{V}}$O_2unloaded(real)_) was 116 mL/min (95% CI=16.4-216.12; Figure 4B). At peak effort, Lin’s concordance coefficient was 0.04 (95% CI=-0.03-0.13; Figure 4C). According to the Bland and Altman model, the mean difference ($\dot{\text{V}}$O_2unloaded(predicted)_−$\dot{\text{V}}$O_2unloaded(real)_) was 890 mL/min (95% CI=238.59-1541.95; Figure 4D).

## Discussion

The Wasserman formula (9) has been routinely used in our institution for the choice of progressive load increment (power) applied to the cycle ergometer during the CPET for healthy individuals and those with CAD. Use of this method is based on an attempt to use a less empirical and subjective method of choosing the intensity of the power ramp to be applied during incremental effort tests on a cycle ergometer.

Tests of short duration with intense power increments generate an insufficient quantity of data, thus impairing their interpretation. In addition, a relatively large proportion of the energy that is generated in these tests is based on anaerobic sources. This fact compromises the response of the O_2_ transport variables and the quality and reliability of the exam because the individual may interrupt the test early because of muscle fatigue. Conversely, long tests with small power increments can prolong the period of effort. In these situations, measurements of O_2_ transport at submaximal effort are also compromised because of early termination of the exam. This termination is due to the discomfort caused by the mouthpiece of the system for metabolic analysis, by discomfort of the cycle ergometer’s seat, lack of motivation, and even muscle fatigue (7,9).

Some investigators have been searching for new methods to accurately estimate the cardiovascular functional reserve, using CPET individualized protocols capable of reaching the maximum aerobic power. Myers et al. (11) developed a questionnaire on daily life activities in elderly subjects that took into consideration chest pain, dyspnea, and fatigue (Veteran’s Specific Activity Questionnaire). Using multivariable analysis, they observed that age and the responses to the questionnaire were able to predict exercise tolerance. They proposed a nomogram that predicted the peak metabolic equivalent and the ramp load increment on a treadmill. In 1996, another group studying patients with heart disease developed a new questionnaire based on daily life activities (12). Using multiple regression analysis, the authors observed that age, height, body mass, and the responses to the questionnaire were able to predict $\dot{\text{V}}$O_2peak_for effort tests on a treadmill. However, most of the studies published in the literature did not detail the choice of the protocol that was used for individualization and performance of the effort tests, as concluded by Huggett et al. (10) in a review of maximum aerobic capacity in elderly people.

The first recommendation on the ideal duration of effort tests needed to reach maximum aerobic power was published in 1983 (3). This controversial study involved only five healthy male volunteers, who performed three incremental effort tests on a treadmill and three tests on a cycle ergometer. The authors concluded that, to obtain the highest $\dot{\text{V}}$O_2max_during an incremental effort test, a load increment needs to be selected that will permit a volunteer to reach the limit of his/her effort tolerance within 10±2 min. Two other studies published in 1998 and 2003 demonstrated that reaching $\dot{\text{V}}$O_2max_is possible in incremental effort tests of prolonged duration (25-26 min) in trained and untrained individuals (13,14). However, slightly longer tests with a mean duration of 28 min resulted in a significantly lower $\dot{\text{V}}$O_2max_compared with tests with a mean duration of 11 min (15). Three subsequent studies (16-18) also reported significantly lower $\dot{\text{V}}$O_2max_values for tests with a mean time to exhaustion of 20-27 min compared with tests lasting 8-12 min.

However, another two studies showed that untrained men and women who were submitted to protocols with a mean time to exhaustion of 6.6-7.4 min were able to reach significantly higher $\dot{\text{V}}$O_2max_values than those who were submitted to protocols with a duration of 8-12 min (17,19). Kang et al. (20) also supported findings that recommend tests of short duration. The authors demonstrated that incremental effort tests lasting approximately 5 min enable $\dot{\text{V}}$O_2max_values to be reached that are similar to those obtained with tests lasting 8-12 min.

Incremental effort tests of short duration can be particularly appropriate for trained individuals because of a greater efficiency in the kinetics of oxygen transport (21). However, these short duration protocols may not be appropriate for patients with cardiorespiratory dysfunction. Agostoni et al. (22) reported significantly higher $\dot{\text{V}}$O_2max_values in protocols of incremental effort with a mean duration of 9.7±0.8 min compared with tests of 5.3±0.5 min in patients with heart failure.

Risk factors for CAD have been documented in men older than 45 years, especially in those with known CAD (23,24). Therefore, the age of subjects in the HG was an average of 10 years less than that of subjects in the CG because most of the risk factors (arterial hypertension, diabetes mellitus, and smoking) excluded HG volunteers. The remaining anthropometric characteristics, including body mass and height, were similar in the two groups.

### HG

HG volunteers were selected to permit inclusion of subjects who were considered “healthy”. However, they could be overweight and/or have dyslipidemia. This criterion was adopted because of the difficulty in finding sedentary men older than 40 years with no regular medications and without risk factors that were considered to be modifiable (25).

In our study, the difference detected between $\dot{\text{V}}$O_2unloaded(predicted)_and $\dot{\text{V}}$O_2unloaded(real)_was only 6%, and did not reach significance. This result indicates that this component of the Wasserman formula may be appropriate for predicting $\dot{\text{V}}$O_2_during the unloaded period in this Brazilian sample of healthy volunteers. However, a significant difference was observed for peak effort, where $\dot{\text{V}}$O_2peak(real)_was 14% lower than $\dot{\text{V}}$O_2peak(predicted)_. In this case, the formula overestimated the real capacity of an individual. Because of this difference, the ability of the formula to predict power increment (ramp) is impaired. Bland and Altman analysis also showed low agreement between the predicted and real $\dot{\text{V}}$O2. There are few data to compare with our results. Recently, another study (26) created and validated a formula that calculates $\dot{\text{V}}$O2peak(predicted) in Brazilian healthy subjects. This previous study also showed that the Wassermann equation may not be suitable in our population.

### CG

Despite the presence of impaired cardiac function, subjects in the CG were unable to achieve maximum effort when the load increment indicated by the Wasserman formula (9) was applied. Subjective adjustments were necessary according to the clinical conditions and degree of physical activity existing at the time of performing the CPET.

The differences detected in the analysis of the CG were of greater magnitude than those in the HG. In the unloaded condition, $\dot{\text{V}}$O_2unloaded(real)_was 18% lower than $\dot{\text{V}}$O_2unloaded(predicted)_, which was a significant difference. For peak effort, this difference was further increased and $\dot{\text{V}}$O_2unloaded(real)_was 40% lower than $\dot{\text{V}}$O_2peak(predicted)_. Therefore, in the CG, the measurements that were predicted by the Wasserman equation always overestimated the real aerobic capacity of the individuals. This fact indicates the need to continue to use a correction factor for power increment (ramp) in subjects with CAD who are evaluated by a routine CPET. Analysis of the agreement between $\dot{\text{V}}$O_2_measurements also showed an even more marked disagreement than that observed in the HG.

Wasserman et al. (9) made an important contribution to scientific knowledge and their work was the basis of our clinical application. However, our results suggest that there is variation related to the experimental design and the individual characteristics involved in the formula.

When we compared the behavior of cardiorespiratory variables during the CPET, we demonstrated a difference in functional capacity between patients with CAD and healthy individuals. The routine use of β-blockers is recommended in patients with CAD. The beneficial effects of this class of medications on improvement of symptom-limited effort capacity have been well established. These effects include a reduction in myocardial ischemia due to effort, an increase in the ischemic threshold, and an improvement in autonomic modulation (27,28).

However, β-adrenoceptor blockade strongly influences metabolic and hemodynamic adaptations and ion balance during dynamic exercise. One of the main effects of the use of a β-blocker is a reduction of heart rate at rest and during dynamic exercise (27-31), as we observed in the present study. A lower heart rate was observed in subjects in the CG during the rest phase and during the various effort phases (unloaded, load increment, and peak effort) compared with subjects in the HG, who did not use medications.

Cardiac output and blood pressure are reduced by the use of β-blockers, although to a lesser extent than heart rate (29-32). According to Pearson et al. (30), this reduction may affect the normal vasoconstriction response in less active territories during exercise because cardiac output, and consequently muscle blood flow, are reduced. Therefore, although there is evidence that muscle fatigue is also caused by neuromuscular mechanisms (33,34), this reduced blood flow may explain, at least in part, early fatigue.

Finally, lower $\dot{\text{V}}$O_2_values were observed in the CG during rest and during the different phases of effort compared with the HG. This reduction, which has already been observed in other studies that evaluated the effect of administration of β-blockers on O_2_ transport (30,31), may partially explain the reduction in $\dot{\text{V}}$O_2_in the CG.

For a prediction or estimation equation, understanding the structural and local characteristics of the population for which the equation is created or validated is important. Predicting $\dot{\text{V}}$O_2peak_is challenging, mainly because factors, such as genetic differences, ethnicity, habits, body size, and physical activity levels of a particular population, may differ from the population in which the equation was initially tested.

### Study limitations

As in other seminal studies (3,9), our study has several limitations. Our sample size was small in both groups, but our strict selection criteria enabled the groups to be uniform.

The present study provides initial evidence that the Wasserman formula does not appear to be appropriate for prediction of functional capacity of Brazilian volunteers, regardless of whether they are apparently healthy or have CAD. Therefore, we cannot precisely predict the power increment (ramp) in incremental CPET on a cycle ergometer. When healthy subjects are compared with those with CAD, the disagreement between measurements is much more marked in the latter than the former.
